# Anti-IgLON5 encephalitis is associated with anti-retinal immunological reactivity without retinal alteration

**DOI:** 10.1016/j.jtauto.2026.100359

**Published:** 2026-02-16

**Authors:** Marie Rafiq, Fanny Varenne, Jérémie Pariente, Fabienne Ory-Magne, Marie Wolfrum, Fleur Gérard, Laurine Virchien, Anne-Laurie Pinto, Bastien Joubert, Françoise Fortenfant, Damien Biotti, Chloé Bost

**Affiliations:** a Department of Neurology, University Hospital of Toulouse, France; b Constitutive Reference Centre for Paraneoplastic Syndromes and Autoimmune Encephalitis, University Hospital of Toulouse, France; c Laboratory Toulouse NeuroImaging Center, INSERM, UMR, 1214, Toulouse, France; d Department of Ophthalmology, University Hospital of Toulouse, Toulouse, France; e French Reference Centre on Paraneoplastic Neurological Syndromes and Autoimmune Encephalitis, Hospices Civils de Lyon, France; f Laboratory of Immunology, Federative Biology Institute, University Hospital of Toulouse, France; g Laboratory INFINTY, INSERM U1043, CNRS UMR 5282, Toulouse, France

**Keywords:** IgLON5 disease, Autoimmune encephalitis, Anti-retinal antibodies, Neuro-ophtalmology, Retina

## Abstract

Anti-IgLON5 disease is a recently defined autoimmune disorder of the central nervous system associated with autoantibodies against IgLON5. This progressive condition, combining features of autoimmunity and neurodegeneration, presents with highly heterogeneous symptoms, including sleep disorders, bulbar symptoms, oculomotor dysfunction, gait disturbances, and subsequent cognitive decline. Recent reports have also described cases of papillitis. The target antigen, IgLON5, is a cell adhesion protein whose role is not fully understood. In humans, it is mainly expressed in the brain and testis. IgLON5 transcripts are also expressed in the retina. However, retinal involvement is not classically explored in these patients. In this cross-sectional observational study, we investigated whether anti-IgLON5 antibodies might target retinal structures, and correlated these findings with ophthalmological assessments. Six patients were diagnosed with anti-IgLON5 antibody encephalitis at Toulouse University Hospital. Identification of the anti-IgLON5 antibody was performed by immunofluorescence on transfected cells using serum and CSF. Anti-retinal antibodies were detected by an indirect immunofluorescence method on sections of monkey retina. Patients underwent a systematic ophthalmological examination including an anatomical and electrophysiological assessment. Anti-retinal antibody identification revealed specific staining of the inner plexiform layer in all patients, which was not observed in control individuals. However, morphological and electrophysiological ophthalmological examinations did not reveal any common features between the patients. Although retinal involvement is rarely reported, these findings suggest a possible role for the retina in the pathophysiology of anti-IgLON5 encephalitis. They support the relevance of considering ophthalmological monitoring in patients with IgLON5-related disease.

## 1 Introduction

Anti-IgLON5 disease is a recently identified autoimmune disorder affecting the nervous system, characterized by autoantibodies directed against the neuronal cell adhesion molecule IgLON5 (gene LOC402665) [1]. Typically diagnosed in individuals in their sixth decade, it presents with a spectrum of chronic or subacute symptoms, often including sleep disturbances such as parasomnias, sleep apnea or excessive daytime sleepiness, as well as gait ataxia, bulbar syndrome, chorea and occasionally cognitive impairment [2]. Oculomotor abnormalities such as vertical/horizontal gaze paralysis, nystagmus or ptosis are also commonly observed [3,4]. Recently, three cases of bilateral papillitis as an initial manifestation of anti-IgLON5 disease have been described in the literature. Two of these patients exhibited additional, commonly observed but moderate features of the disease, including balance disturbances and mild cognitive impairment, while one case presented with isolated papillitis [5,6]. Finally, a patient was described with a disease initially manifested by recurrent episodes of optic neuritis associated with obstructive sleep apnea syndrome, daytime hypersomnolence and abnormal movements [7]. To date, ophthalmological involvement has been documented in only these four cases, and post-mortem studies have not investigated ocular or retinal damage in anti-IGLON5 encephalitis.

Immunologically, blood-brain barrier disruption and pleocytosis in the cerebrospinal fluid (CSF) have been observed [8], anti-IgLON5-IgG4 predominate, and a notable correlation with the HLA-DRB1∗10:01 allele has been described [9]. Although the precise role of IgG anti-IgLON5 antibodies remains unclear, they appear to play a central role in the pathology, potentially inducing long-term neuroinflammation and neuronal damage [10]. Interestingly, the retina is the second most abundant organ expressing IgLON5 RNA, after the brain and before the testis, according to the transcriptome database^1^ [11]. Furthermore, the presence of RNA transcripts of the IgLON5 protein was identified by RT-PCR in the mouse nervous system, predominantly in the brainstem (the pons and the medulla oblongata), the thalamus and the eyes [12]. However, there are no reports on the protein expression of IgLON5 in the human eye.

We met a 68-year-old patient with a history of obstructive sleep apnea who developed a progressive neurological syndrome including gait impairment, bilateral ptosis, oculomotor and bulbar dysfunction, trismus and chorea ultimately diagnosed as anti-IgLON5 disease two years after onset. She reported recent visual acuity loss, and ophthalmological examination revealed bilateral central serous chorioretinopathy with macular detachment, more pronounced in the left eye. Given this atypical presentation, we investigated a possible link between anti-IgLON5 antibodies and retinal involvement, drawing on our laboratory's expertise in autoimmune retinopathy. The discovery of specific retinal immunoreactivity in this patient prompted us to assess retinal antibody binding in all patients diagnosed for anti-IgLON5 encephalitis at the University Hospital of Toulouse, France, using both serum and CSF samples. In parallel, we conducted systematic ophthalmological and electrophysiological evaluations to detect potential subclinical retinal involvement.

## 2 Method

### 2.1 Patients and data collection

All patients enrolled exhibited clinical symptoms consistent with anti-IgLON5 encephalitis and demonstrated the presence of anti-IgLON5 antibodies in serum and cerebrospinal fluid with a concordant staining on cerebellum slices (ref. 504225e Inova Diagnostics), confirmed on cell-based assay (CBA ref. FA1151-1005-50, Euroimmun).

The study encompassed all patients diagnosed with anti-IgLON5 encephalitis at Toulouse University Hospital, France. Clinical data were gathered from patients' medical records, including demographic information (gender, age, medical history), age of symptom onset, duration between symptom onset and diagnosis, total disease duration at the time of the study, mode of presentation (subacute/chronic), clinical manifestations of the disease, findings from cerebral MRI, cerebrospinal fluid and blood sample analyses, treatments administered, and clinical course.

### 2.2 Detection of anti-retinal antibodies

The reactivity of the patients’ sera and/or CSF against the retina was evaluated retrospectively with frozen samples (−80°, EXPLAINEUR biobank) through an indirect immunofluorescence technique using sections of monkey retina (Ref. FA1172-1005, Euroimmun), detected with an FITC-labelled secondary antibody anti-human IgAGM (Euroimmun conjugate) or directed against IgA (ref. F0204, DAKO), IgM (ref. F0203, DAKO), IgG1 (ref. 9052-02, Southern Biotech) and IgG4 (ref. 9200-02, Southern Biotech). Non-human primate retina was used due to its close anatomical and molecular similarity to the human retina, including a conserved laminar organization and neuronal stratification [13], and because more than 90% of human retinal cell types have transcriptomically corresponding counterparts in macaque, with largely conserved expression of disease-related genes across species [14]. This assay is a standardized commercial test routinely used for several years in our laboratory for clinical purposes. All slides were read independently by two experienced investigators (C.B. and F.F.). The test has been validated locally through analysis of a large series of control samples, including sera from 238 patients with autoimmune or non-autoimmune ophthalmologic diseases, 40 patients with other autoimmune disorders, including neurological conditions, and 10 healthy controls.

### 2.3 Immunoadsorption assay

To confirm the specificity of the immunofluorescence pattern observed on monkey retina, immunoadsorption was performed using HEK293 cells transfected with human IgLON5. CSF and serum from patient 1 were diluted 1:100 and 1:5000, respectively. Each diluted sample was successively incubated in wells containing live IgLON5-transfected HEK293 cells (16 wells for CSF and 17 wells for serum) to allow specific antibody binding and depletion. The supernatants collected after the final passage were then re-evaluated by indirect immunofluorescence on monkey retina sections to assess for residual antibody reactivity.

### 2.4 Ophthalmological investigations

#### 2.4.1 Clinical examination

The patients included were also examined systematically in the ophthalmology unit. An anatomical assessment was performed using a combined spectral domain (SD), high-resolution optical coherence tomography (OCT) with a scanning laser ophthalmoscope (SPECTRALIS® HRA + OCT, Heidelberg Engineering, Heidelberg, Germany). Macular SD-OCT was performed in all patients using a single line horizontal scan (ART [Automated Real Time] 100 frames) passing through the fovea. N = 4/6 patients (identifications 3, 4, 5 and 6) also achieved a volume scan of 25 multicross sections in horizontal direction and centered on the fovea (ART 9 frames), with a slice angle of 20° to measure inner plexiform layer thickness which was reported in an Early Treatment of Diabetic Retinopathy Study (ETDRS) macular map. Only the 1-mm diameter ring scan was considered for the analysis and defined as central thickness. A scan of the optic disc was also performed, including a 3.5-mm diameter ring scan of the peripapillary retinal nerve fiber layer (pRNFL).

#### 2.4.2 Testing procedure

Full-field electroretinography (ffERG) was performed according to guidelines from the International Society for Clinical Electrophysiology of Vision (ISCEV Standard ERG protocol, Robson, 2022) using a Ganzfeld apparatus (Vision Monitor, Métrovision, Pérenchies, France) and contact sterile thread DTL electrode. Peak times and wave amplitudes were measured according to guidelines from ISCEV and compared with our laboratory-specific reference values for dark-adapted (DA) ERG responses to flash intensities (in photopic units; phot) of 0.01, 3 and 10 phot cd-s-m-2 (DA 0.01; DA 3; DA 10) and for light-adapted (LA) ERG responses to single flash intensities of 3 phot cd-s-m-2 (LA 3) and at a frequency close to 30 Hz (LA 30 Hz). Only qualitative analysis was carried out for DA oscillatory potentials (OPs), generated by the 3 cd-s-m-2 flash stimuli (DA 3 OPs), in accordance with ISCEV recommendations.

#### 2.4.3 Ethics

All patients included in this study were enrolled in the EXPLAINEUR clinico-biological collection (protocol number 2020-A02169-30). The study was approved by the Comité de Protection des Personnes (CPP) Tours–Ouest I, France, and was conducted in accordance with applicable national regulations and ethical standards. All participants provided written informed consent prior to inclusion.

## 3 Results

### 3.1 Clinical manifestations

A total of seven patients were diagnosed with anti-IgLON5 antibody encephalitis at Toulouse University Hospital, France between 2019 and 2023. However, only six patients (three men and three women) met the inclusion criteria for this study. The detailed clinical characteristics of these patients are provided in Table 1. The median age at inclusion was 72 years (range: 63–81), with symptoms manifesting at a median age of 68 years (range: 61-79). Half of the patients exhibited a subacute symptom onset (within 6 months), while the other half had a chronic presentation. The median anti-IgLON5 disease composite score (ICS) (Caig, 2024) at onset was 14/69 [11.5-19.5]. In terms of neuro-ophthalmological findings, three patients reported visual symptoms. These included horizontal-rotatory nystagmus in one patient, a complex oculomotor disorder with bilateral ptosis and decreased visual acuity in the initial patient with a central serous chorioretinopathy, and a gaze disorder with unilateral ptosis in a third patient. Brain MRI scans were normal in all patients except one, who exhibited moderate cerebellar atrophy. The treatment protocol is described in Table 1. Despite treatment, three patients experienced progressive symptom deterioration, resulting in the death of one patient due to respiratory failure. The median ICS at last visit was unchanged at 14/69 [11.5-19.5] (Table 1).

**Table 1:** **Patient characteristics**. IVIG: intravenous immunoglobulin; RTX: Rituximab; CYC: Cyclophosphamide; PE: Plasma Exchange. ICS: Anti-IgLON5 Disease Composite Score (/69).

Patient	Sex	Age (y)	Age at onset (y)	Presentation	ICS at onset	Time to diagnosis (y)	Disease duration (y)	Subacute/Chronic onset	Abnormal ocular movements	Brain MRI	CSF analysis	Treatment	Outcome	ICS at last visit
**Patient 1**	M	74	68	Sleep disorder (apnea and excessive daytime sleepiness), gait instability	15	4	6	Chronic	No	Non specific change	Normal	IVIG; RTX; CYC	No change	15
**Patient 2**	F	72	67	Gait instability	7	2	5	Chronic	Horizontal and torsional nystagmus	Mild cerebellar atrophy	Normal	IVIG; RTX; CYC	No change	7
**Patient 3**	F	72	68	Cognitive complaints, gait instability and hallucinations	11	2	4	Chronic	No	Non specific change	Elevated tau and phospho-tau and decreased Abeta 1-42	IVIG; RTX	Progressive cognitive decline	14
**Patient 4**	M	81	79	Cognitive complaints, gait instability and abnormal movements	21	1	1	Subacute	No	Non specific change	High protein level	IVIG; PE; RTX; CYC	Worsening of gait instability and abnormal movements	24
**Patient 5**	F	71	68	Bulbar symptoms, gait instability, abnormal movements	24	2	3	Subacute	Complex oculomotor palsy and bilateral ptosis	Non specific change	Normal	IVIG; PE; RTX; CYC	Gradual worsening until death by respiratory failure	NA
**Patient 6**	M	63	61	Abnormal ocular movements and headaches	13	1	2	Subacute	Vertical gaze palsy and right ptosis	Non specific change	Pleocytosis (20 cells) and high protein level	RTX; CYC	Moderate improvement	6

### 3.2 Biological investigations

Cerebrospinal fluid (CSF) analysis revealed that two patients had hyperproteinorachia, while one patient showed mild pleocytosis, with 20 white blood cells per microliter. None of the patients exhibited intrathecal oligoclonal bands or an elevated IgG and/or kappa index.

### 3.3 Indirect immunofluorescence staining on retinal slices

**Fig. 1:**
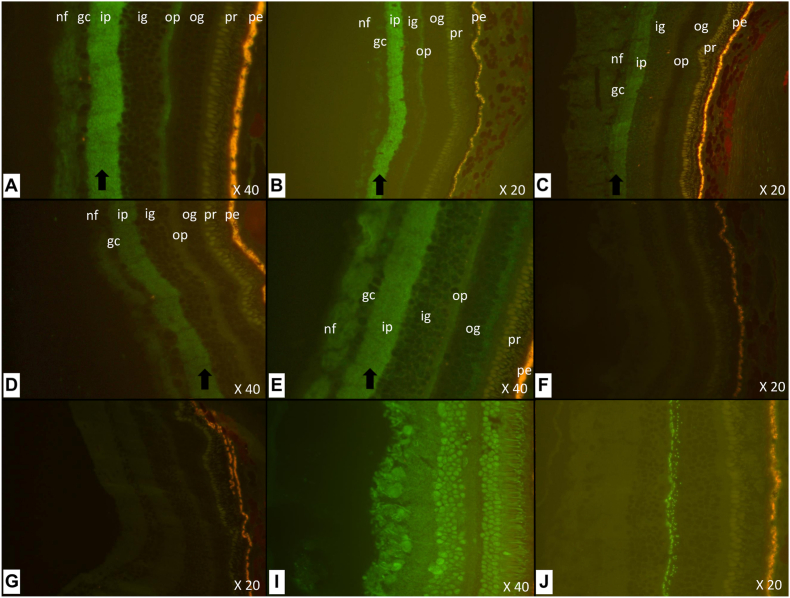
**Indirect immunofluorescence on sections of monkey retina.** The fluorescence observed is identified by a black arrow. (A) Patient 4 (IgG4); (B) Patient 1 (IgG4); (C) Patient 3 (IgG1); (D) Patient 5 (IgG4); (E) Patient 2 (IgG1); (F) Patient 1 (IgG4) after immunoadsorption of IgLON5 antibodies; (G) Control with macular edema (IgG1); (H) Control with anti-Hu encephalitis (IgG1); (I) Control with CAR syndrome (IgG4). The different layers of the retina are identified by their initials: pigment epithelium (pe), photoreceptor layer (pr), outer grain layer (og), outer plexiform layer (op), inner grain layer (ig), inner plexiform layer (ip), ganglion cell layer (gc), nerve fibre layer (nf).

Indirect immunofluorescence analysis of retinal tissue revealed a distinct staining of the inner plexiform layer in all six patients tested (Fig. 1). Fluorescence was observed using both anti-human IgG1 and IgG4 secondary antibodies, while no signal was detected with anti-IgM or anti-IgA. Interestingly, most patients were double positive for both IgG1 and IgG4 subclasses. However, two patients were only positive with anti-IgG1, resulting in a higher number of IgG1-positive cases (5/6) compared to IgG4 (4/6), despite the generally stronger fluorescence signal observed with anti-IgG4. This staining pattern was not observed in any of the samples previously analyzed within Immunology laboratory, which included healthy individuals, patients with autoimmune retinopathies (paraneoplastic or non-paraneoplastic), individuals with other non-autoimmune ocular disorders and patients with autoimmune encephalitis or paraneoplastic neurological syndrome (as illustrated in Fig. 1). Importantly, the staining was no longer detectable after immunoadsorption of anti-IgLON5 antibodies from the CSF of patient 1 (Fig. 1 f).

### 3.4 Ophthalmological explorations

**Fig. 2:**
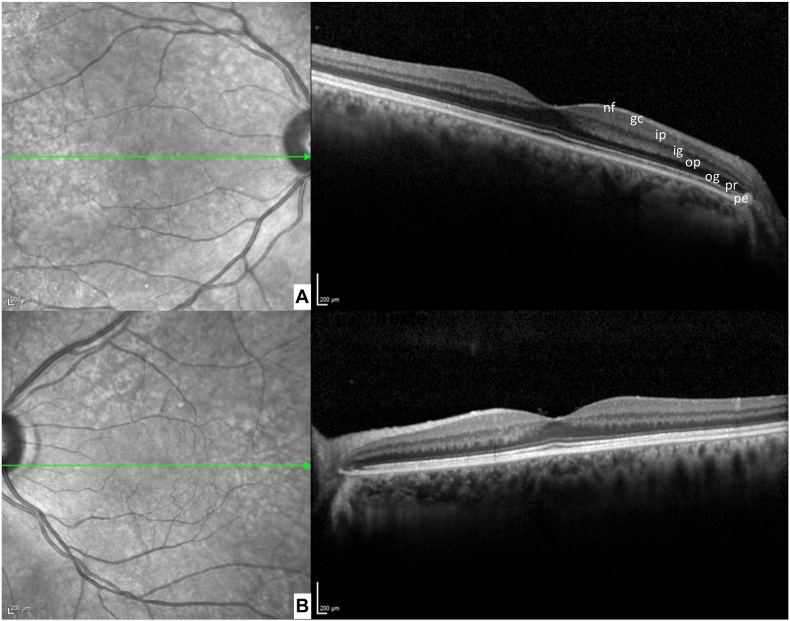
Macular SD-OCT of Patient 1. (A) Right eye. (B) Left eye. The different layers of the retina are identified by their initials: pigment epithelium (pe), photoreceptor layer (pr), outer grain layer (og), outer plexiform layer (op), inner grain layer (ig), inner plexiform layer (ip), ganglion cell layer (gc), nerve fibre layer (nf).

Apart from the three patients with neuro-ophthalmological no evidence of systematic ophthalmological repercussions was identified by ophthalmological explorations in any of the 6 patients explored. The results from the anatomical and electrophysiological assessments are presented in Table 2. Anatomically, no apparent abnormalities were observed on macular SD-OCT, notably in the inner plexiform layer (Fig. 2). No cases developed macular edema. The thickness of the inner plexiform layer in the 1-mm ring scan could be measured reliably in 3 patients (identifications 3, 4 and 6); mean thicknesses were 21.67 μm in the right eye and 22 μm in the left eye, within the lower range of published normative data. Two patients had macular SD-OCT abnormalities independent of autoimmune disease; one patient with right tilted disc syndrome and bilateral epiretinal membrane, and the other patient had a known history of left eye chronic central serous chorioretinopathy (Patient 5). On peripapillary SD-OCT, only two patients exhibited moderate to severe optic atrophy (reduced pRNFL thickness in at least one retinal quadrant). In addition, we did not observe any optic disc edema.

**Table 2:** **Results of neuro-ophthalmological investigations.** pRNFL: peripapillary retinal nerve fibers layer. IPL: Inner Plexiform Layer. RE: Right Eye. LE: Left Eye. ETDRS: Early Treatment Diabetic Retinopathy Study.

Patient	Thickness of IPL in 1 mm ETDRS macular map (μm)	Macular SD-OCT	RNFL	ffERG
**Patient** 1	No macular cube available for measurement	Papillary dysversion syndrome on the right, bilateral epiretinal membrane	Severe global optic atrophy on the left and uninterpretable on the right (papillary dysversion)	Normal
**Patient** 2	No macular cube possible due to nystagmus	Normal foveal profile	No RNFL measurement possible	Normal
**Patient** 3	20/23	Normal foveal profile	Normal	Normal
**Patient** 4	27/24	Normal foveal profile	Normal	Normal
**Patient** 5	Measurement biased by alterations to the retina, not taken into account	Macular drusen on the right; pigment epithelial detachment and retinal serous detachment on the left	Moderate overall optic atrophy	Normal
**Patient** 6	18/19	Normal foveal profile	Normal	Normal

Electrophysiological assessments showed that all full-field electroretinograms (ffERG) were within normal limits. Specifically, the peak times and amplitudes of the a- and b-waves in both photopic single-flash and flicker responses were within our laboratory's reference values. The b-to-a wave amplitude ratios (b/a ratios) were greater than 1 in all patients for scotopic responses recorded after 3.0 cd s/m^2^ (DA 3.0) and 10.0 cd s/m^2^ (DA 10.0) dark-adapted flashes. Additionally, all patients exhibited at least three distinguishable peaks in the oscillatory potentials (OPs) recorded under DA 3.0 conditions.

## 4 Discussion

All six patients with anti-IgLON5 encephalitis included in this study exhibited a consistent and selective immunoreactivity of their antibodies in the inner plexiform layer of monkey retinal tissue, with no staining in other retinal layers. This specific and reproducible pattern supports a targeted interaction between anti-IgLON5 antibodies and retinal structures, which had not been previously described.

These findings align with transcriptomic and anatomical data suggesting the presence of IgLON5 protein in the retina. RNA expression of IGLON5 has been reported in the eye in animal models (Protein Atlas; [12]), and *in situ* hybridization studies in mice have revealed IgLON5 transcript expression in the ganglion cell layer and inner nuclear layer during prenatal development [15]. As IgLON5 is a membrane-bound protein, its localization within the inner plexiform layer—adjacent to these layers—is plausible. Anatomically, the inner plexiform layer corresponds to synapses between bipolar and ganglion cells, and is composed of amacrine cells involved in signal integration, particularly in the rod pathway.

This specific labelling pattern distinguishes anti-IgLON5 antibody reactivity from that seen in other autoimmune retinopathies. For instance, anti-recoverin antibodies preferentially target the photoreceptor layer [16], while in melanoma-associated retinopathy, immunoreactivity typically involves the inner nuclear, outer plexiform, and nerve fiber layers [17]. Moreover, this pattern was not observed in any of the other participants tested in our laboratory, including healthy controls and patients with either ophthalmological or other autoimmune disorders.

The disappearance of the retinal signal following immunoadsorption with anti-IgLON5 antibodies further supports the specificity of this reactivity. While retinal immunostaining cannot replace standardized antibody testing, it may help confirm diagnoses as a complementary diagnostic tool in challenging situations and raises the question of whether ophthalmological monitoring should be more systematically considered in these patients. Recent reports of papillitis in anti-IgLON5 patients suggest that subclinical retinal or optic nerve involvement may be underrecognized. Based on these findings, a comprehensive ophthalmological examination appears advisable in patients with anti-IgLON5 encephalitis, particularly in the presence of visual complaints or abnormal findings, and longitudinal follow-up could be considered when initial abnormalities are detected.

Despite the detection of this fluorescence for all patients examined, no association between inner plexiform layer staining and anatomical/electrophysiological retina impairment was found. Indeed, ffERG recording enables the distinction between outer and inner retinal dysfunction. The absence of anomalies therefore confirms the proper functioning of the cone and rod system, notably rod and cone bipolar cells that synapse in the inner plexiform layer. Furthermore, although not yet fully elucidated, the cellular origins of OPs on ffERG appear to reflect inner retinal activity involving amacrine cells and retinal ganglion cells. The presence of distinct oscillatory potentials (OPs) on ffERG in all our patients suggests the integrity of the inner retina, and in particular of the amacrine cells located in the inner plexiform layer. We attempted to perform a pattern ERG (pERG) in all of our patients to better determine if ganglion cells synapsing in the inner plexiform layer were impaired [18]. Indeed, pERG arises largely in the ganglion cells, driven by the photoreceptors and corresponding retinal cells. However, we did not obtain reliable results in the vast majority of cases due to fixation difficulties and loss of ocular transparency (e.g., corneal opacities, cataract) in this elderly population with neurological disorders.

Furthermore, no anatomical abnormalities of the macula were found and the inner plexiform layer had normal reflectivity on SD-OCT without irregularities. Although measured only in 3 patients, the average central thickness of the inner plexiform layer was comparable to reports in the literature in a healthy Caucasian population aged 69 to 87 years (mean 21.7 μm, 1st percentile 13 μm, 5th percentile 16 μm and 95th percentile 28.8 μm) [19]. The optic atrophy observed in two patients could reflect damage to ganglion cells, whose synapses with amacrine and bipolar cells are located in the inner plexiform layer. However, it is impossible to assert a causal link with anti-IgLON5. On the one hand, because there were no pRNFL abnormalities for the other patients despite identical immunofluorescence marking. On the other hand, the causes of optic atrophy vary widely. Given the absence of prior pRNFL data for these patients, the optic atrophy could have developed before the encephalitis.

While anti-IgLON5 antibodies have been shown to be directly pathogenic [10,20,21], patients carrying these antibodies do not exhibit retinal abnormalities. This apparent discrepancy—despite *in vitro* binding of the antibodies to primate retinal tissue—may reflect their limited access to the retina *in vivo*, possibly due to protective barriers such as the blood-retinal or blood-CSF barriers. Moreover, interindividual variability in the timing of sample collection—ranging from the acute to the chronic phase of the disease—may further limit the ability to detect retinal involvement, particularly in studies aiming to elucidate pathogenic mechanisms.

It can therefore be surmised that anti-IgLON5 antibodies, or the plasma cells, may traverse the blood-brain barrier (BBB) and contribute to neurological symptoms, while failing to cross the blood-retinal barrier (BRB) in most cases [11,22]. Indeed, the BRB comprises two distinct components: an outer and an inner barrier. The outer BRB consists of junctions between retinal pigment epithelium cells, regulating hydro-ionic and metabolic exchanges between the choriocapillaris and the outer retina. The inner BRB (iBRB), partly located in the inner plexiform layer due to the retinal intermediate capillary plexus, is formed by tight junctions between endothelial cells of non-fenestrated retinal capillaries, along with pericytes and the end-feet of retinal macroglial cells. This inner barrier forms a neurovascular unit structurally and functionally analogous to the BBB [23].

Compared to the BBB, retinal endothelial cells display the smallest intercellular spaces and the highest number of tight junction strands among all endothelial cells, which may explain why anti-IgLON5 antibodies or their producing immune cells can cross the BBB but not the iBRB.

The absence of macular edema on spectral-domain optical coherence tomography (SD-OCT) in our patients indirectly supports the integrity of the BRB. However, only fluorescein angiography could have confirmed BRB integrity through the absence of dye leakage—an investigation that was not performed in our cohort.

The limited number of patients precludes the possibility of generalizing the immunological observations made on the retina. It is conceivable that in some patients with anti-IgLON5 encephalitis, the blood-retinal barrier may be crossed, leading to inflammatory changes in the retina. This hypothesis could explain the recent observations of patients presenting with bilateral neuropapillitis or optic neuritis in the context of anti-IgLON5 disease, with no other etiology identified, and responding favourably to immunosuppressive treatment [[5], [6], [7]].

## 5 Conclusions

In this study, we observed consistent binding of anti-IgLON5 antibodies to the inner plexiform layer of the retina using an indirect immunofluorescence technique, suggesting a possible expression of IgLON5 in this region that has not been clearly characterized so far. Interestingly, this reactivity was not associated with any clear anatomical or electrophysiological abnormalities in our patients, though similar cases have begun to appear in the literature. One possible explanation is that the structure of the blood-retinal barrier, which differs from the blood-brain barrier, may limit access of circulating antibodies to retinal tissue. Finally, our findings underline the difficulty in understanding the mechanisms of these rare antibody-mediated diseases, where tissue expression of the target antigen does not necessarily lead to functional impairment, but may still give rise to symptoms depending on immunological factors that remain poorly understood.

## CRediT authorship contribution statement

**Marie Rafiq:** Writing – review & editing, Writing – original draft, Supervision, Project administration, Methodology, Investigation, Formal analysis, Data curation, Conceptualization. **Fanny Varenne:** Writing – review & editing, Methodology, Investigation, Formal analysis, Data curation, Conceptualization. **Jérémie Pariente:** Writing – review & editing, Investigation, Formal analysis. **Fabienne Ory-Magne:** Investigation, Formal analysis. **Marie Wolfrum:** Investigation, Formal analysis. **Fleur Gérard:** Investigation, Formal analysis. **Laurine Virchien:** Writing – review & editing. **Anne-Laurie Pinto:** Methodology, Investigation. **Bastien Joubert:** Writing – review & editing, Methodology, Investigation. **Françoise Fortenfant:** Writing – review & editing, Methodology, Investigation, Formal analysis, Data curation, Conceptualization. **Damien Biotti:** Writing – review & editing, Formal analysis, Conceptualization. **Chloé Bost:** Writing – review & editing, Supervision, Methodology, Investigation, Formal analysis, Data curation, Conceptualization.

## Funding

Immunoabsorption studies were performed within the BETPSY project, which is supported by a public grant overseen by the French national research agency (Agence nationale de la recherche, 10.13039/501100001665ANR), as part of the second “Investissements d'Avenir” program (reference ANR-18-RHUS-0012).

## Declaration of competing interest

The authors declare that they have no known competing financial interests or personal relationships that could have appeared to influence the work reported in this paper.

## Data Availability

Data will be made available on request.

## List of abbreviation

CSF: Cerebrospinal fluid
ICS: Anti-IgLON5 disease Composite Score
OCT: Optical Coherence Tomography
OP: Oscillatory Potential
ffERG: full-field Electroretinography
BRB: Blood-Retinal barrier
BBB: Blood-Brain Barrier
RNFL: Retinal Nerve Fiber Layer

## Footnotes

1 https://www.proteinatlas.org/ENSG00000142549-IGLON5/tissue.
